# Potential safety signal of pregnancy loss with vascular endothelial growth factor inhibitor intraocular injection: A disproportionality analysis using the Food and Drug Administration Adverse Event Reporting System

**DOI:** 10.3389/fphar.2022.1063625

**Published:** 2022-11-10

**Authors:** Takamasa Sakai, Chiyo Mori, Fumiko Ohtsu

**Affiliations:** ^1^ Drug Informatics, Faculty of Pharmacy, Meijo University, Nagoya, Japan; ^2^ Angelbells Hospital, Okazaki, Japan

**Keywords:** pregnancy loss, pharmacovigilance, signal detection, spontaneous reporting databases, VEGF inhibitors

## Abstract

**Objectives:** Intraocular administration of vascular endothelial growth factor (VEGF) inhibitors may be associated with pregnancy loss. However, little is known about intraocular anti-VEGF therapy during pregnancy. Here, we conducted a pharmacovigilance study using a spontaneous reporting database to evaluate the relationship between intraocular VEGF inhibitors and pregnancy loss.

**Methods:** We used the JAPIC AERS database which is composed of the Food and Drug Administration Adverse Event Reporting System (FAERS) dataset preprocessed by the Japan Pharmaceutical Information Center (JAPIC) to investigate the VEGF inhibitors ranibizumab, aflibercept, and bevacizumab. Disproportionality analyses were conducted for VEGF inhibitors and pregnancy loss. The lower limit of the 95% confidence interval (CI) of the reporting odds ratio (ROR) > 1 and a minimum of three reported cases of pregnancy loss were the detection criteria used in the current study.

**Results:** In the FAERS, 19 pregnancy loss cases were reported for ranibizumab with an ROR of 4.44 (95% CI: 2.42–8.16), 6 for intraocular bevacizumab with an ROR of 32.25 (95% CI: 3.88–267.9), and 4 for intraocular aflibercept with an ROR of 5.37 (95% CI: 1.34–21.49). All these drugs met the detection criteria.

**Conclusion:** Potential safety signals of pregnancy loss were obtained from intraocular administration of VEGF inhibitors during pregnancy. These signals should be validated using a causal design study.

## Introduction

Intraocular anti-vascular endothelial growth factor (VEGF) therapy is commonly used because of its efficacy in various diseases such as choroidal neovascularization, retinal vascular occlusion, and diabetic retinopathy/macular edema. All these diseases are seen in not only older adult patients but also women of childbearing age, meaning that pregnant patients could be exposed to these drugs without awareness of their pregnancy (Kianersi et al., 2016; Naderan et al., 2021). Intraocular administration of VEGF inhibitors such as ranibizumab, aflibercept, and bevacizumab has been shown to enter systemic circulation and reduce VEGF levels in the blood (Avery et al., 2014). VEGF contributes to vascular shape and function (Bautch, 2012), and both VEGF-A polymorphisms and reduced VEGF expression have been associated with spontaneous miscarriage, likely owing to defective fetal and placental angiogenesis (Almawi et al., 2013). Because human immunoglobin G is known to cross the placental barrier, anti-VEGF antibodies may also cross the placenta. Therefore, intraocular administration of VEGF inhibitors may be associated with pregnancy loss. However, only a few studies based on a small number of case reports have examined the association between VEGF inhibitors and adverse pregnancy outcomes, and the results are controversial (Polizzi and Mahajan, 2015; Naderan et al., 2021). Further evidence is required, but the number of cases at individual medical institutions is limited.

Spontaneous reporting is a fundamental source of information in pharmacovigilance. Usually, the risk of adverse events from drug exposure during pregnancy is examined in study designs involving a control group, such as in cohort studies. However, these studies require a sufficient number of cases and may require significant time and effort to conduct. To obtain safety signal data for small study populations earlier, some pharmacovigilance researchers have begun using the spontaneous reporting database (Deepak and Stobaugh, 2014; Sakai et al., 2017; Sessa et al., 2019; van De Ven et al., 2020). An international survey of pharmacovigilance centers has also reported intentions to implement or improve spontaneous reporting for drug exposure during pregnancy (Kant et al., 2019). Lareb, a pharmacovigilance center in the Netherlands, has discussed the use of a spontaneous reporting database to develop a toolkit for drug safety surveillance in pregnant women (Lareb, 2018).

Here, we conducted a pharmacovigilance study using a large spontaneous reporting database to evaluate the relationship between intraocular VEGF inhibitors and pregnancy loss.

## Materials and methods

### Data source

We collected data from the Food and Drug Administration (FDA) Adverse Event Reporting System (FAERS), a spontaneous reporting database containing records from pharmaceutical companies, medical institutions, and consumers. Data collected were split into seven tables reporting these essential categories: patient demographic information (DEMO), drug information (DRUG), indications for the use of reported drugs (INDI), therapy start and end dates (THER), adverse events (REAC), adverse event outcomes (OUTC), and report sources (RPSR). The cases included in this study were reported from the fourth quarter of 1997 to the fourth quarter of 2021. The FAERS dataset version used was processed by the Japan Pharmaceutical Information Center (JAPIC). They removed duplicate cases, such as those with the same “CASEID,” leaving the non-duplicated cases. In the dataset, drug names were mapped to generic names using their respective drug name dictionaries; reported adverse events and indication of use were assigned to their preferred term (PT) codes and the age was converted to years. The drug name dictionary is based on the World Health Organization drug dictionary and Drugs@FDA (https://www.accessdata.fda.gov). For drug names not included in these dictionaries, dictionaries were created based on the Summary of Product Characteristics for each product, as well as on drug information databases such as Martindale and MIMS (https://www.mims.com/). This preprocessed FAERS dataset (JAPIC AERS) was coded in the Medical Dictionary for Regulatory Activities (MedDRA) ver 25.0, which we used for analysis in the current study.

### Pregnancy-related report retrieval

Subgroup disproportionality analyses are used to control for possible bias when analyzing the relationship between drugs and pregnancy outcomes in datasets in which most reports are from non-pregnant women (Beyer-Westendorf et al., 2020; Huybrechts et al., 2021). However, there is no established algorithm for identifying reports of pregnant women from spontaneous reporting databases (Deepak and Stobaugh, 2014; Sessa et al., 2019; Sandberg et al., 2020; Sakai et al., 2022). To our knowledge, no regulatory authority has provided any specific guidance for such procedure. Because the spontaneous reporting databases do not usually contain a dedicated field to identify reports of pregnant women, measures are being taken to identify such reports using the standard MedDRA query (SMQ) (Sessa et al., 2019; Sakai et al., 2022). Free text and author-specific keyword searches other than dictionaries have also been conducted, but their reliability is unknown (Deepak and Stobaugh, 2014). Therefore, in the current study, we used our previously described method to identify reports of pregnant women in the FAERS (Sakai et al., 2022).

**FIGURE 1:**
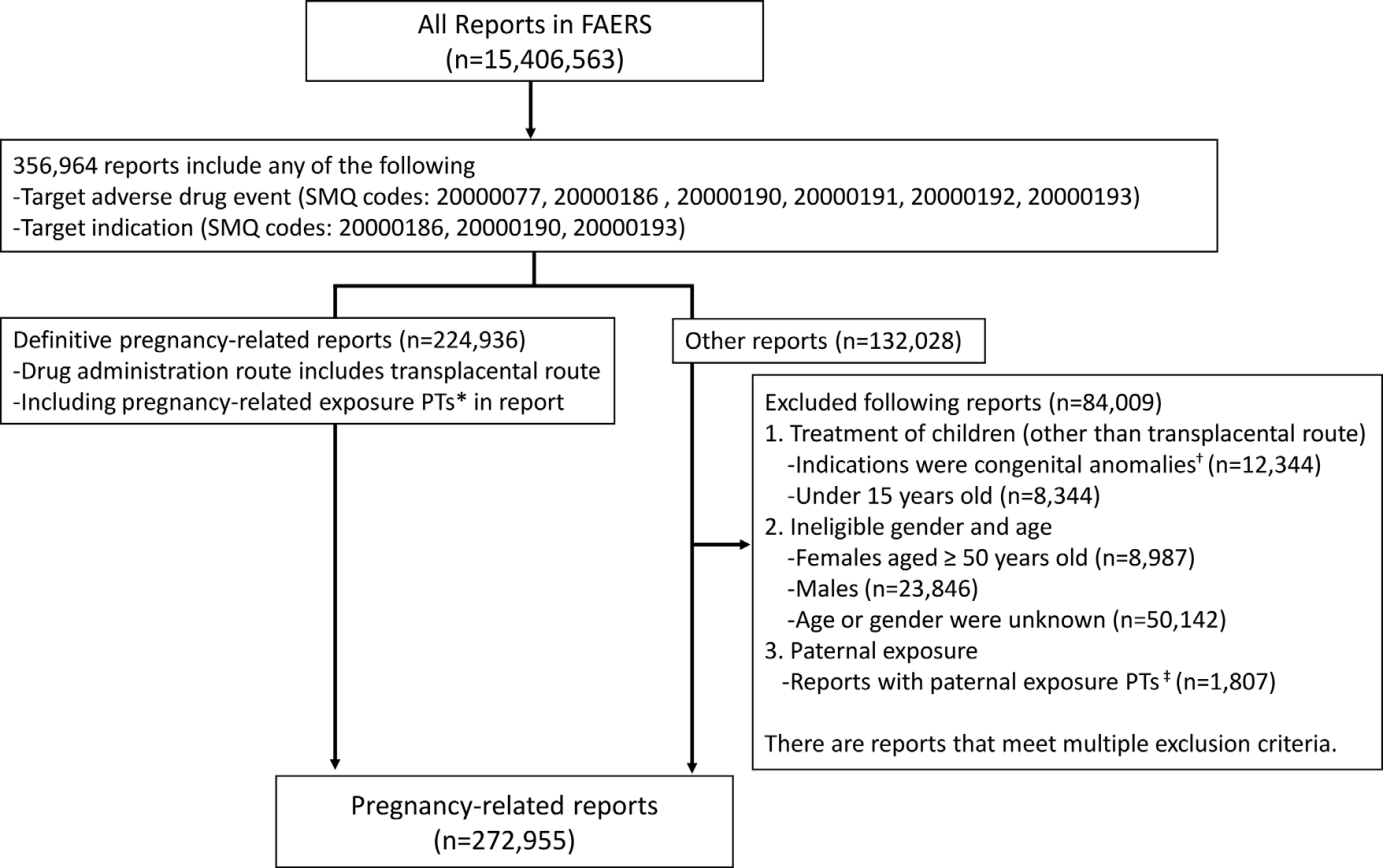
Data extraction of pregnancy-related reports from the Food and Drug Administration Adverse Event Reporting System. *PTs: maternal exposure during delivery (10071407), foetal exposure during delivery (10071409), maternal exposure before pregnancy (10071406), maternal exposure during pregnancy (10071408), fetal exposure during pregnancy (10071404), exposure during pregnancy (10073513), maternal exposure timing unspecified (10071415), foetal exposure timing unspecified (10071405), maternal drugs affecting foetus (10026923), drug exposure before pregnancy (10064998). ^†^SMQs: congenital, familial, and genetic disorders (20000077). ^‡^PTs: paternal drugs affecting the fetus (10050425), exposure *via* father (10071403), paternal exposure during pregnancy (10080091), paternal exposure timing unspecified (10080092), paternal exposure before pregnancy (10080093), and maternal exposure *via* partner during pregnancy (10084938). PT, Preferred term; SMQ, standard MedDRA query.

All sub-SMQs of the SMQ “pregnancy and neonatal topics,” except for “lactation related topics (including neonatal exposure through breast milk)” were utilized to identify candidate cases for pregnancy-related reports. Based on these reports, cases that contained pregnancy-related exposure PTs in Figure 1 or where the route of administration was transplacental were defined as definitive pregnancy-related reports. Except for definitive pregnancy-related reports, we excluded cases with patients of ineligible sex and age, along with those of paternal exposure (Figure 1). The reports obtained through these procedures were considered as pregnancy-related reports.

### Investigated drugs and adverse events

Drugs under investigation included the VEGF inhibitors ranibizumab, aflibercept, and bevacizumab. Because aflibercept and bevacizumab can be administered both intraocularly and intravenously, we included only reports of intraocular administration. Cases with an unknown route of administration were excluded. Drugs in the FAERS database were categorized into four drug roles: “primary suspect drug,” “secondary suspect drug,” “concomitant,” and “interacting,” depending on their level of involvement in the expected adverse events. Only reports of “primary suspect drug” or “secondary suspect drug” were included in this analysis.

Regarding adverse events, we initially analyzed the number of reports of the PT included in the SMQ “termination of pregnancy and risk of abortion,” which includes adverse events that are unlikely to have been caused directly by the drug, such as induced abortion and infectious miscarriage. As a result, a definition of pregnancy loss was created that excluded these adverse events, and the remaining terms were used as target adverse events (Table 1).

**TABLE 1:** Definitions of pregnancy loss in this study.

PT code	PT name
10000209	Aborted pregnancy
10000210	Abortion
10000212	Abortion complete complicated
10000217	Abortion incomplete
10000218	Abortion incomplete complicated
10000230	Abortion missed
10000234	Abortion spontaneous
10000236	Abortion spontaneous complete complicated
10000238	Abortion spontaneous complicated
10000239	Abortion spontaneous incomplete complicated
10042062	Stillbirth
10052846	Abortion early
10052847	Abortion late
10055690	Fetal death
10061614	Abortion complete
10061615	Abortion complicated
10061616	Abortion spontaneous complete
10061617	Abortion spontaneous incomplete

Abbreviation: PT, preferred term.

### Disproportionality analysis

**FIGURE 2:**
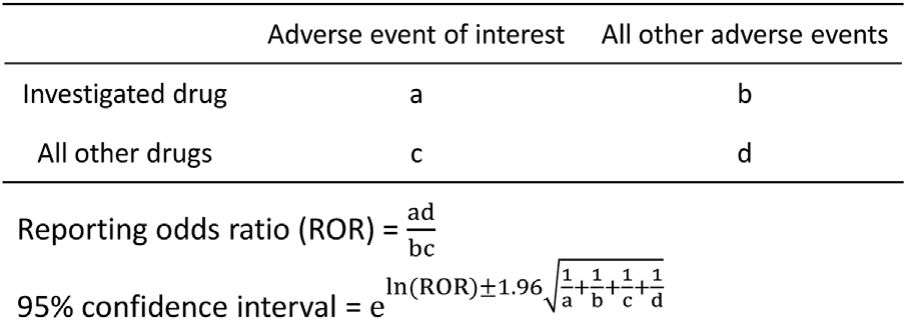
Two by two contingency table and the reporting odds ratio formula.

We created two by two contingency tables from pregnancy-related reports and calculated reporting odds ratios (RORs) (Figure 2). The detection criteria were defined as the lower limit of the 95% confidence interval of the ROR > 1 under the condition that a minimum of three cases of the target adverse event were reported, as per the criteria of the European Medicines Agency (Wisniewski et al., 2016; Sessa et al., 2019). We also conducted two sensitivity analyses, one using the PT “abortion spontaneous” as the only target adverse event, and one using definitive pregnancy-related reports. Statistical analyses were performed using the open-source R software (version 4.2.1).

### Review of individual case safety reports

For the drugs where signals were detected, individual safety reports were reviewed to further assess the relationship. The survey items included age, drug, indication, adverse events, and reporting period. Drugs were checked to see if any have been shown to be harmful during pregnancy with the Australian Therapeutic Goods Administration classification. We also checked for duplicate reports that could not be excluded by preprocessing.

## Results

### Extraction of pregnancy-related reports

A total of 272,955 reports of pregnant women were extracted from the FAERS. Pregnancy loss was reported in 42,821 cases. Among pregnancy-related reports, 42 involved ranibizumab, 8 involved intraocular aflibercept, and 7 included intraocular bevacizumab. The reported adverse events for the SMQ “termination of pregnancy and risk of abortion” for each drug are shown in Table 2. Among these events, the PT “abortion spontaneous” was the most reported, and fewer than three “stillbirth” PTs were reported for any of the investigated drugs.

**TABLE 2:** Adverse events related “Termination of pregnancy and risk of abortion (SMQ)”.

Drug name	N
Ranibizumab	*n* = 42
Abortion spontaneous	12
Abortion	2
Fetal death	2
Stillbirth	2
Abortion induced	2
Abortion missed	1
Aflibercept (intraocular)	*n* = 8
Abortion spontaneous	2
Abortion missed	1
Stillbirth	1
Bevacizumab (intraocular)	*n* = 7
Abortion spontaneous	6

### Disproportionality analysis

Pregnancy loss was reported in 19 cases with ranibizumab, 6 cases with intraocular bevacizumab, and 4 cases with intraocular aflibercept (Table 3). Ranibizumab met the detection criteria with an ROR of 4.44 (95% CI: 2.42–8.16), as did the sensitivity analysis results. Intraocular bevacizumab also met the detection criteria with an ROR of 32.25 (95% CI: 3.88–267.9), as did the sensitivity analysis results. Intraocular aflibercept also met the detection criteria with an ROR of 5.37 (95% CI: 1.34–21.49), but the sensitivity analysis results did not meet the criteria.

**TABLE 3:** Number of reports and reporting odds ratio of pregnancy loss.

drug	Pregnancy loss			Sensitivity analysis 1: PT “abortion spontaneous” only			Sensitivity analysis 2: Pregnancy loss (restricted to definitive pregnancy-related reports)		
	Case (n)	Total (n)	ROR [95% CI]	Case (n)	Total (n)	ROR [95% CI]	Case (n)	Total (n)	ROR [95% CI]
Ranibizumab	19	42	4.44 [2.42–8.16]	12	42	3.38 [1.73–6.61]	19	40	5.30 [2.85–9.87]
Bevacizumab (intraocular)	6	7	32.25 [3.88–267.9]	6	7	50.71 [6.11–421.28]	4	5	23.44 [2.62–209.77]
Aflibercept (intraocular)	4	8	5.37 [1.34–21.49]	2	8	2.82 [0.57–13.96]	3	7	4.40 [0.98–19.64]

Abbreviations: CI, confidence interval; PT, preferred term; ROR, reporting odds ratio.

### Review of individual case safety reports

The data from individual cases of pregnancy loss with ranibizumab, intraocular bevacizumab, or intraocular aflibercept treatment are shown in Tables 4, 5, and 6, respectively. In each case, no suspected drugs were classified as X by the Australian Therapeutic Goods Administration (TGA) classification. Some cases reported concomitant use of ranibizumab and azathioprine, which holds an Australian TGA D classification.

**TABLE 4:** Individual case safety reports of pregnancy loss with ranibizumab.

No	Age	Drug	Indication	Adverse event	Report year and quarter
1	NA	Ranibizumab (PS)	Pseudoxanthoma elasticum	Abortion spontaneous	2008 Q1
2	NA	Ranibizumab (PS)	Product used for unknown indication	Abortion spontaneous	2010 Q3
3	NA	Ranibizumab (PS)	Product used for unknown indication	Abortion spontaneous	2011 Q2
4	28	Ranibizumab (PS)	Choroidal neovascularisation	Abortion spontaneous, Chorioretinal disorder, Visual acuity reduced	2011 Q3
5	33	Ranibizumab (PS/SS)	Retinal vein occlusion	Abortion	2011 Q4
6	26	Ranibizumab (PS)	Diabetic retinal oedema	Abortion spontaneous	2014 Q2
7	46	Ranibizumab (PS/SS), ACTOVEGIN™ (C), Ofloxacin (C)	Retinal vein thrombosis	Abortion spontaneous	2014 Q2
8	27	Ranibizumab (PS), Dexamethasone (C)	Retinal vein thrombosis, Retinal vein occlusion	Abortion spontaneous	2015 Q1
9	NA	Ranibizumab (PS/SS), Levothyroxine sodium (C)	Pathologic myopia, Choroidal neovascularisation	Abortion spontaneous	2015 Q4
10	NA	Ranibizumab (PS/SS)	Diabetic retinopathy, Diabetic retinal edema	Abortion spontaneous	2015 Q4
11	42	Ranibizumab (PS/SS)	Macular oedema	Abortion	2016 Q2
12	NA	Ranibizumab (PS)	Product used for unknown indication	Abortion spontaneous	2016 Q2
13	34	Ranibizumab (PS)	Product used for unknown indication	Abortion spontaneous	2018 Q4
14	NA	Prednisolone (PS), Ranibizumab (SS), Azathioprine (SS)	Chorioretinitis, Antiangiogenic therapy	Premature separation of placenta, Stillbirth, Product use in unapproved indication	2021 Q2
15	NA	Ranibizumab (PS/SS), Prednisolone (SS), Azathioprine (SS)	Chorioretinitis, Choroiditis, Antiangiogenic therapy	Premature separation of placenta, Stillbirth, Product use in unapproved indication	2021 Q2
16	NA	Azathioprine (PS), Ranibizumab (SS), Prednisolone (SS)	Choroiditis	Premature separation of placenta, Foetal death	2021 Q2
17	NA	Azathioprine (PS), Ranibizumab (SS), Prednisolone (SS)	Choroiditis	Premature separation of placenta, Foetal death, Product use in unapproved indication	2021 Q2
18	32	Ranibizumab (PS/SS)	Choroidal neovascularisation	Abortion missed	2021 Q3
19	38	Ranibizumab (PS)	Diabetic retinal oedema	Abortion spontaneous	2021 Q3

Abbreviations: C, Concomitant; NA, not available; PS, primary suspect drug; SS, secondary suspect drug. Pregnancy-related exposure preferred terms are omitted.

**TABLE 5:** Individual case safety reports of pregnancy loss with intraocular bevacizumab.

No	Age	Drug	Indication	Adverse event	Report year and quarter
1	29	Bevacizumab (PS), Insulin (C)	Diabetic retinopathy, Type 1 diabetes mellitus	Abortion spontaneous	2009 Q3
2	29	Bevacizumab (PS)	Choroidal neovascularisation	Abortion spontaneous	2009 Q3
3	25	Bevacizumab (PS)	Product used for unknown indication	Abortion spontaneous	2009 Q3
4	39	Bevacizumab (PS)	Choroidal neovascularisation	Abortion spontaneous, Off label use	2014 Q1
5	36	Bevacizumab (PS/SS), Propranolol (C), Omeprazole (C), Venlafaxine (C), Cholecalciferol (C), CRANBERRY EXTRACT (C)	Presumed ocular histoplasmosis syndrome	Fetal growth restriction, Hemorrhage, Abortion spontaneous, Off label use	2018 Q2
6	25	Fluorescein sodium (PS), Bevacizumab (SS), Indocyanine green (C)	Angiogram retina, Choroidal neovascularisation	Abortion spontaneous	2018 Q2

Abbreviations: C, Concomitant; NA, not available; PS, primacy suspect drug; SS, secondary suspect drug. Pregnancy-related exposure preferred terms are omitted.

**TABLE 6:** Individual case safety reports of pregnancy loss with intraocular aflibercept.

No	Age	Drug	Indication	Adverse event	Report year and quarter
1	47	Aflibercept (PS), Folic acid (C)	Retinal vein occlusion	Abortion missed	2014 Q3
2	NA	Aflibercept (PS/SS)	Diabetic retinal oedema	Abortion spontaneous. Product use issue	2016 Q3
3	NA	Aflibercept (PS/SS)	Diabetic retinal oedema	Abortion spontaneous, Diabetes mellitus inadequate control	2017 Q1
4	NA	Aflibercept (PS)	Diabetic retinopathy	Stillbirth, Abnormal weight gain, Abnormal loss of weight, Malaise, Renal disorder, Respiratory disorder, Cardiac failure	2017 Q3

Abbreviations: C, Concomitant; NA, not available; PS, primacy suspect drug; SS, secondary suspect drug. Pregnancy-related exposure preferred terms are omitted.

## Discussion

Our findings present safety signals of pregnancy loss after administration of some VEGF inhibitors. Among pregnancy loss cases, the PT “abortion spontaneous” was the most reported adverse event. Pregnancy loss in this study refers primarily to spontaneous abortion, not stillbirth. Because PT level signal detection is also a common method for analyzing safety signals (Wisniewski et al., 2016), we conducted a disproportionality analysis using only the PT “abortion spontaneous” as a sensitivity analysis. The number of PT “stillbirth” events reported was fewer than three for all VEGF inhibitors, meaning that this PT did not meet the detection criteria. Therefore, we did not conduct a disproportionality analysis using only the PT “stillbirth” as a sensitivity analysis.

VEGF plays an essential role in both fetal and placental angiogenesis. Some reports suggest a link between VEGF expression level and recurrent miscarriages (Vuorela et al., 2000). Therefore, the use of a VEGF inhibitor treatment could theoretically cause pregnancy loss, owing to the reduction of the plasma level of free VEGF (Avery et al., 2014).

Ranibizumab is a protein encoding a Fab fragment of the VEGF-A antibody; the effect of the absence of the Fc site on placental transfer is unknown, and no information on placental transfer in humans is available (Briggs et al., 2021). The effect of ranibizumab on plasma VEGF concentrations is weaker than other VEGF inhibitors and is eliminated earlier (Zehetner et al., 2013; Avery et al., 2014). Although it appears to be safer for use during pregnancy than other VEGF inhibitors, experience with its use in pregnant women is limited. Ranibizumab pertains to the Australian TGA “D” classification, indicating an association with adverse effects. Three case reports showed no adverse outcomes observed after intraocular ranibizumab administration in the third trimester (Sarhianaki et al., 2012; Jouve et al., 2015). For exposure during early pregnancy, one report indicated that miscarriage occurred 6 d after exposure (Akkaya, 2019), whereas other reports indicated no adverse outcomes (Fossum et al., 2018). In this study, we performed disproportionality analyses on a larger number of cases than in previous studies using the spontaneous reporting database, allowing for consistent signal detection and enabling sensitivity analysis. We believe that our results support the hypothesis that ranibizumab is associated with pregnancy loss. Detailed analysis of individual cases showed that the patients’ age ranged from 26 to 46 years, and pregnancy loss was not necessarily associated with older age. Based on reports No. 14–17 in Table 4, the possibility that the same case was reported in duplicate cannot be ruled out, although the FAERS, PRIMARYID (a unique number for identifying a FAERS report), and CASEID (a number for identifying a FAERS case) identifiers were different. In addition, these suspected duplicate reports may have been influenced by the concomitant use of azathioprine, which is categorized as “D” by the Australian TGA. However, the safety signal was still detected when these four cases were excluded; therefore, the presence of these reports does not significantly affect the results of this study.

Bevacizumab is a recombinant humanized monoclonal IgG antibody of VEGF that was approved for the treatment of metastatic colorectal cancer and is now also being used as an off-label therapeutic for eye diseases (Polizzi and Mahajan, 2015). Bevacizumab has been reported to reduce plasma VEGF concentrations for at least 1 month (Zehetner et al., 2013; Avery et al., 2014). However, this drug was associated with the most case reports of exposure during pregnancy, and Naderan et al. (2021) argued that it is used more for pregnant women than other VEGF inhibitors. In this study, only a limited number of cases was obtained specifically for intraocular administration. However, six of the seven cases were associated with pregnancy loss, and the signal was consistently detected, including in sensitivity analyses. Cases of miscarriage have been reported following intraocular bevacizumab administration during early pregnancy (Petrou et al., 2010; Gómez Ledesma et al., 2012). Our results support the conclusions of these previous studies that intraocular bevacizumab administration is associated with pregnancy loss. On the contrary, a study reported using single injections during early pregnancy without adverse outcomes (Sullivan et al., 2014). However, it remains unclear whether bevacizumab causes spontaneous abortion. Counseling is recommended to disclose the off-label nature of the treatment and to explain both its efficacy and the potential risks to pregnant patients.

Aflibercept is a recombinant fusion protein comprising the extracellular domains of VEGF receptors 1 and 2 fused to the Fc domain of human IgG and binds to VEGF and placental growth factor. Because of its higher affinity, aflibercept is reported to reduce plasma VEGF concentrations the most among the three VEGF inhibitors investigated in this study (Avery et al., 2014; Polizzi and Mahajan, 2015). Studies using animal models have exhibited external, skeletal, and visceral malformations after intravenous aflibercept administration during pregnancy. It is categorized as D in the Australian TGA classification. Intraocular aflibercept (EYLEA™) is contraindicated for pregnant women in Japan, and embryo-fetotoxicity is listed as a potential risk in the European risk management plan (European Medicines Agency, 2022). In this study, a safety signal of pregnancy loss was detected for intraocular aflibercept. However, the number of cases was limited, and the sensitivity analysis did not meet the signal detection criteria. To our knowledge, no published case reports currently describe human aflibercept exposure during pregnancy; therefore, we consider this finding to be an important signal.

The current study had several limitations derived from using the spontaneous reporting database (Sakai et al., 2020; Noguchi et al., 2021). First, it is well known that signals from disproportionality analysis frequently show false positive results owing to the effects of reporting biases (Wisniewski et al., 2016). Second, the lack of denominator information in the spontaneous reporting database, make us impossible to calculate the incidence rate of pregnancy loss. The ROR results do not necessarily reflect the risk intensity. Owing to the limited number of cases, statistical analysis was unstable, showing a wide range of ROR values. Third, limited information is available on individual case safety reports (Tsuchiya et al., 2020). Age, history of miscarriage (Magnus et al., 2019), alcohol consumption, smoking, and obesity (Magnus et al., 2022) are risk factors for miscarriage. Age was obtained in some reports, but data for other factors were not available. However, the findings provided evidence of important safety signals regarding the association between VEGF inhibitors and miscarriage, even though limited case reports were available until now. Given that signal detection is a hypothesis-generating design study, future efforts to collect cases and confirm/disprove hypotheses with well-designed comparative safety studies will greatly contribute to this investigation.

## Data Availability

Publicly available datasets were analyzed in this study. This data can be found here: https://fis.fda.gov/extensions/FPD-QDE-FAERS/FPD-QDE-FAERS.html.
